# Kenneth C. Holmes (1934–2021)

**DOI:** 10.1107/S1600577522002211

**Published:** 2022-02-25

**Authors:** John Wray, Ilme Schlichting

**Affiliations:** a Max Planck Institute for Medical Research, Jahnstrasse 29, 69120 Heidelberg, Germany

## Abstract

Obituary for Kenneth C. Holmes

Ken Holmes was born in England and studied physics at St John’s College, Cambridge, from where he graduated in 1955. His initial interest in crystallography led him to apply to the Department of J. D. Bernal at Birkbeck College, London (part of London University). Bernal was a pioneer of research on the molecular structure of living material, which triggered Ken’s specialization in diffraction by fibrous materials. 
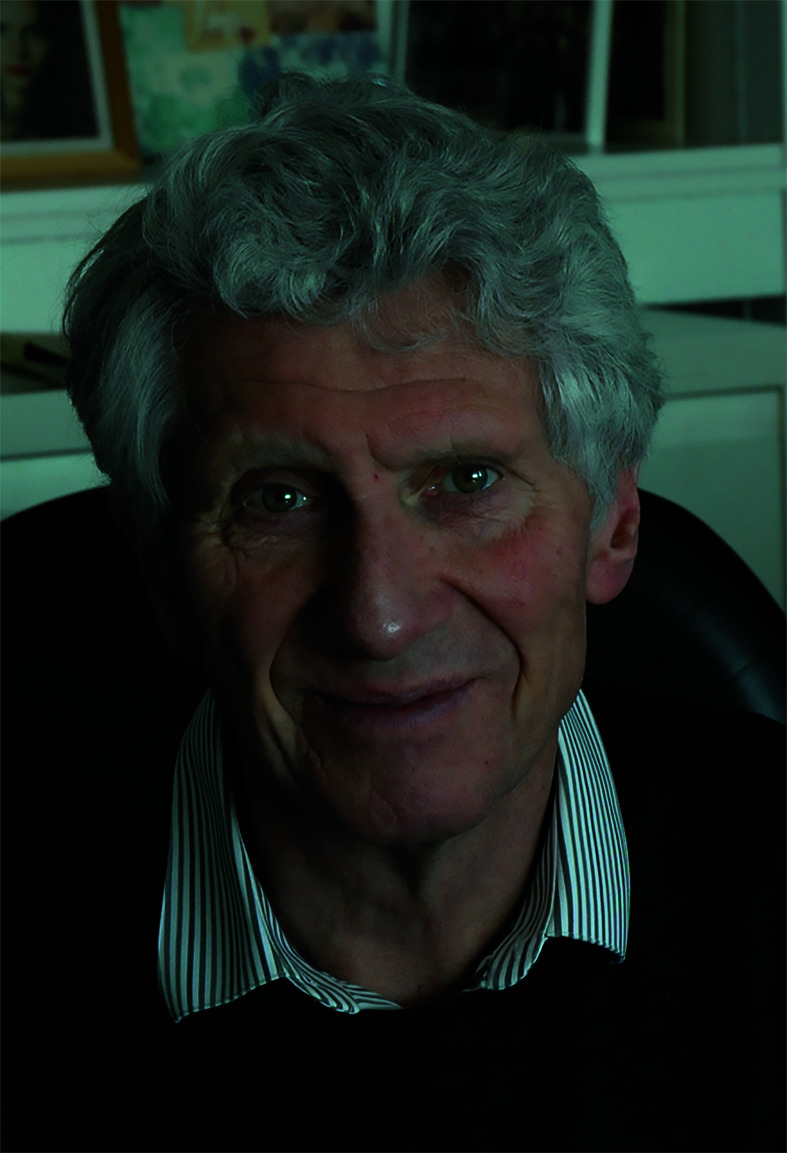


In 1955 he started his PhD under Rosalind Franklin. His thesis work focused on the tobacco mosaic virus (TMV), for which Bernal and Fankuchen had, as early as 1936, found clear evidence of remarkable regularity using X-ray diffraction. This regularity was known to be that of a helix; as Ken noted many years later, this was not quite what he had understood by crystallography! His work focused on the equator of the pattern and, in a first use of isomorphous replacement with a fibrous structure, obtained an image of the projection down the helix axis. Aaron Klug had already joined Franklin’s group in 1953, and Don Caspar soon became a regular visitor there also. Aaron and Don became life-long friends for Ken, and Don died last year, only a few weeks after Ken.

Franklin died in 1958, and Ken finished his PhD under Klug’s leadership in 1959. He and his wife Mary then spent 15 months in Boston. Ken had planned to work there with Don Caspar at the Children’s Hospital, but his main project ended up being with Don’s colleague Carolyn Cohen on the structure of paramyosin in the ABRM: Ken’s first introduction to muscle. Ken and Mary returned to England in time for the birth of their first child. Ken received an appointment at the relatively new MRC lab in Cambridge, where Hugh Huxley had for some years been studying the structure of muscle, and the central goal was soon to obtain diffraction patterns from living muscle fibres. This work focused on the construction of X-ray sets with rotating anodes to allow greater intensity. In 1963 Mike Reedy arrived as a postdoc, and he and Ken, together with Richard Tregear, started an investigation of insect flight muscle: intact but permeablized. This preparation was ideally suited for studying structure (by X-ray and EM), mechanics and biochemistry and led to a major breakthrough: the finding that the appearance of the ‘crossbridges’ depended on the absence or presence of ATP, the energy source for contraction. By the mid-1960s, Ken was looking for a permanent position, and he was considered for a directorship at the Max Planck Institute for Medical Research in Heidelberg. He had had contact with the MPI for Virology in Tübingen, and the idea of Heidelberg was attractive. He knew, moreover, that one of the world’s first synchrotrons was in Hamburg.

Ken was duly appointed and moved to Heidelberg in September 1968. His projects there continued to concern diffraction from fibres, namely muscle filaments and the TMV particle. The crystallography of TMV was progressing in Cambridge: disk-like aggregates of the protein subunit had been found, allowing the structure of the subunit to be solved crystallographically. The structure of the native helix was pursued (with Stubbs and Warren) using further derivatives, leading finally to a refined structure of the native rod-shaped particle. The work of Ken’s department progressed in several other directions: developing more intense X-rays for observing changes in structure during contraction, the structure of the actin and myosin filaments of striated muscle, and the structural analysis of other proteins which, like myosin, split nucleotides.

Ken’s move to Heidelberg immediately opened a window of opportunity regarding intense X-ray sources. His student Gerd Rosenbaum had studied at DESY in Hamburg and knew the situation there – and support was available from the DESY directors, who sensed that the prospect of biological applications would raise further support for DESY. (A few years later, the situation reversed: Ken’s initiative at the DESY outstation made Heidelberg an attractive site for the newly founded EMBL.) Ken and Gerd spent long periods at DESY and finally established the world’s first beamline there. Beamlines at synchrotrons world-wide followed from this work, and the resultant advances in crystallography have since then revolutionized structural biology.

The second challenge was the crystallography of actin. The native actin filament resembled TMV in being a non-integral helix, but was variable and gave fibre diffraction patterns with too little resolution for determination of the atomic structure. Its globular monomer moreover resolutely refused to form crystals, but did so as a complex with DNAse. The analysis of this involved great practical difficulties, but the structures of the actin monomer and filament were finally obtained in 1990.

Work in Ken’s department had involved EM approaches to the structure of myosin also, and ultrastructural studies of nucleotide analogs had raised tantalizing glimpses of a possible structural basis of the force-producing event in muscle. It was a minor sensation when, in 1993, a first X-ray crystallographic structure of subfragment 1 of myosin, the ‘head’ of the molecule and the ‘crossbridge’ in muscle, became available from others. The challenge now was less in crystallography than in the interpretation of the results, and Ken was crucially involved in the fitting of the structure into EM images of actin filaments with myosin attached. Within a few years, several groups had presented clear evidence for polymorphism of the head structure, and intense discussions with several groups converged on a consensus of how myosin and its essential partners (ATP, its hydrolysis products, and actin) produce force and movement. Ken provided a crucial insight that the original S1 structure had been incompletely interpreted and, in reality, presaged the essential changes in myosin’s conformation – and thus the mechanism of force-generation in muscle.

Ken retained the physicist’s eye for essential questions and crucial simplifications. He enjoyed ruthlessly ignoring details and was always ready to abandon well trodden paths when (or even before) the need arose. He died on 2 November 2021. His energy, enthusiasm, clarity and inventiveness will be greatly missed.

